# Rapid, early, and potent Spike-directed IgG, IgM, and IgA distinguish asymptomatic from mildly symptomatic COVID-19 in Uganda, with IgG persisting for 28 months

**DOI:** 10.3389/fimmu.2023.1152522

**Published:** 2023-03-16

**Authors:** Jennifer Serwanga, Violet Ankunda, Jackson Sembera, Laban Kato, Gerald Kevin Oluka, Claire Baine, Geoffrey Odoch, John Kayiwa, Betty Oliver Auma, Mark Jjuuko, Christopher Nsereko, Matthew Cotten, Nathan Onyachi, Moses Muwanga, Tom Lutalo, Julie Fox, Monica Musenero, Pontiano Kaleebu, The COVID-19 Immunoprofiling Team, Patricia Namubiru

**Affiliations:** ^1^ Pathogen Genomics, Phenotype, and Immunity Program, Medical Research Council, Uganda Virus Research Institute and London School of Hygiene and Tropical Medicine, Uganda Research Unit, Entebbe, Uganda; ^2^ Department of Immunology, Uganda Virus Research Institute, Entebbe, Uganda; ^3^ Department of Virology, Uganda Virus Research Institute, Entebbe, Uganda; ^4^ Department of Internal Medicine, Masaka Regional Referral Hospital, Masaka, Uganda; ^5^ Department of Internal Medicine, Entebbe Regional Referral Hospital, Entebbe, Uganda; ^6^ Medical Research Council, University of Glasgow Centre for Virus Research, Glasgow, United Kingdom; ^7^ Department of Epidemiology and Data Management, Uganda Virus Research Institute, Entebbe, Uganda; ^8^ Guy’s and St Thomas’ National Health Services Foundation Trust, King’s College London, London, United Kingdom; ^9^ Science, Technology, and Innovation Secretariat, Office of the President, Government of Uganda, Kampala, Uganda

**Keywords:** SARS-CoV-2 antibody persistence, Spike and RBD, nucleoprotein, mild and asymptomatic COVID-19, IgG, IgM, IgA, Uganda

## Abstract

**Introduction:**

Understanding how spike (S)-, nucleoprotein (N)-, and RBD-directed antibody responses evolved in mild and asymptomatic COVID-19 in Africa and their interactions with SARS-CoV-2 might inform development of targeted treatments and vaccines.

**Methods:**

Here, we used a validated indirect in-house ELISA to characterise development and persistence of S- and N-directed IgG, IgM, and IgA antibody responses for 2430 SARS-CoV-2 rt-PCR-diagnosed Ugandan specimens from 320 mild and asymptomatic COVID-19 cases, 50 uninfected contacts, and 54 uninfected non-contacts collected weekly for one month, then monthly for 28 months.

**Results:**

During acute infection, asymptomatic patients mounted a faster and more robust spike-directed IgG, IgM, and IgA response than those with mild symptoms (Wilcoxon rank test, p-values 0.046, 0.053, and 0.057); this was more pronounced in males than females. Spike IgG antibodies peaked between 25 and 37 days (86.46; IQR 29.47-242.56 BAU/ml), were significantly higher and more durable than N- and RBD IgG antibodies and lasted for 28 months. Anti-spike seroconversion rates consistently exceeded RBD and nucleoprotein rates. Spike- and RBD-directed IgG antibodies were positively correlated until 14 months (Spearman’s rank correlation test, p-values 0.0001 to 0.05), although RBD diminished faster. Significant anti-spike immunity persisted without RBD. 64% and 59% of PCR-negative, non-infected non-contacts and suspects, exhibited baseline SARS-CoV-2 N-IgM serological cross-reactivity, suggesting undetected exposure or abortive infection. N-IgG levels waned after 787 days, while N-IgM levels remained undetectable throughout.

**Discussion:**

Lower N-IgG seroconversion rates and the absence of N-IgM indicate that these markers substantially underestimate the prior exposure rates. Our findings provide insights into the development of S-directed antibody responses in mild and asymptomatic infections, with varying degrees of symptoms eliciting distinct immune responses, suggesting distinct pathogenic pathways. These longer-lasting data inform vaccine design, boosting strategies, and surveillance efforts in this and comparable settings.

## Introduction

In 2019, a new human coronavirus illness (COVID-19) caused by the severe acute respiratory syndrome coronavirus 2 (SARS-CoV-2) appeared, sparking a serious public health crisis. By September 2022, there were 613,410,796 COVID-19 cases, including 6,518,749 deaths, and 12,659,951,094 vaccine doses administered (https://covid19.who.int, accessed September 29, 2022). Of these, 9,327,413 cases and 174,509 deaths occurred in sub-Saharan Africa (SSA), revealing a considerably lower impact in SSA (1). Several hypotheses were proposed to explain this lesser disease burden, including a younger demographic structure in SSA (2), less testing, undercounting of deaths, genetic predispositions, and cross-reactive immunity against previous coronaviruses. Pre-existing cross-reactive immune responses have been reported in many geographical locations (3–6) and in some cases were significantly higher in SSA than in other continents (4), probably due to the high sequence homology between SARS-CoV-2 and the common coronaviruses in SSA. Such cross-reactive immune responses to other coronaviruses were linked to a decreased likelihood of COVID-19 disease severity in the United States (7), but not in other regions, such as SSA (8–10).

The Spike (S) protein of SARS-CoV-2 is composed of the S1 and S2 subunits. A receptor-binding domain (RBD) within the S1 subunit interacts with human host cells expressing ACE2 receptors to promote viral entry (11). Antibodies against RBD block virus interaction with the host cell receptors, thus providing protection (12). Accordingly, antibodies directed against the S protein, particularly the RBD, are critical targets for developing vaccines and therapeutics (13–15) due to their positive associations with viral neutralisation titres (16–19). On the other hand, the Nucleoprotein serves as the primary target in many serosurveillance test systems, and serological responses to N infer prior SARS-CoV-2 exposure (20–22).

It is essential to examine the dynamics of humoral immune responses to SARS-CoV-2 to infer protective immunity and determine vaccination-induced immunity. However, the dynamics of the anti-SARS-CoV-2 antibody response and persistence after infection are still debatable and have primarily been studied in the context of more severe disease, which is uncommon in African patients. While antibody persistence was associated with severe disease, comparable seropositivity was reported between symptomatic and asymptomatic individuals in some contexts (23, 24) but not in others (25). Mild COVID-19 disease has been linked to a weaker humoral response, raising fears of faster waning of immunity. Severe disease has been associated with longer persistence of humoral immunity for 12 months post-infection (26, 27). Some populations have shown delayed onset of S-IgG and IgM, making early serological screening less significant (28). Median anti-Spike titres in symptomatic and hospital-admitted cases are significantly higher than in asymptomatic participants, persisting for at least one year. There is a need to establish the dynamics of antibody development in SSA settings where the disease impact has been distinctively different.

It is vital to monitor changes in S-, RBD-, and N-directed IgM, IgG, and IgA levels in sub-Saharan Africa to guide diagnostic strategies, public health policy, and immunological correlates pertinent to vaccine formulation. Multiple viral proteins (29, 30) elicit prompt and long-lasting immunity that persists for several months (31–33). SARS-CoV-2-directed S- and N-IgG, -IgM, and -IgA antibody profiles have guided inference of the serological response to COVID-19 and provided insight into the relevance of targeting the Spike-protein for vaccine design (34, 35). Using data from European cohorts, mathematical modellers predicted the persistence of functional Spike and RBD-directed antibodies 465 days post-infection and faster decay of N-directed antibodies, providing data that inform vaccination and serosurveillance strategies (36). Nevertheless, there is limited knowledge on the development, kinetics, and profile of immune responses to the milder and asymptomatic COVID-19 epidemic that primarily occurred in the African setting. Geographically relevant data is needed to inform vaccination, diagnostic and surveillance strategies in this setting.

On March 21, 2020, Uganda confirmed her first COVID-19 case in a returning traveller, prompting a countrywide lockdown to prevent further spread, except for cargo truck drivers and vital front-line professionals required to safeguard the economy and combat the epidemic, respectively. All inbound cargo truck drivers were mandated to undergo cross-border PCR testing for COVID-19, and all detected cases were quarantined in designated referral hospitals until viral clearance was confirmed by PCR. This approach allowed for possibility of assembling a cohort of newly infected PCR-confirmed COVID-19 cases to profile the local immune response to the epidemic. Consequently, we examined the profile, timing, durability, specificity, seroconversion rates, and targets of humoral immunity to SARS-COV-2 over 28 months in PCR-confirmed COVID-19 convalescent participants with or without re-exposure, as well as the influence of gender and symptom status on induced antibody responses.

## Materials and methods

### Study design and population

A prospective cohort of rt-PCR-confirmed SARS-CoV-2 positive, slightly symptomatic, and asymptomatic participants and rt-PCR-confirmed negative, exposed, and unexposed persons was established. The cohort was established in response to the mandatory requirement for the purposive sampling of all incoming travellers for COVID-19 PCR positivity, while the rest of the country as under lock down. All PCR-positive cases were subjected to mandatory isolation at Masaka and Entebbe Referral Hospitals until they were deemed PCR-negative. Participants were recruited through regional referral hospitals in Entebbe and Masaka, which served as COVID-19 isolation and treatment centres at the start of the Ugandan outbreak. Available hospital records from the participant’s admission were used to obtain participant-related health information. Access to participant-related health information was only possible through available hospital admission records. When possible, an acute blood sample was obtained to assist the COVID-19 care team in obtaining a complete blood count (CBC) report. In some cases, records of conditions such as hypertension, diabetes, and asthma were also collected to better understand the participants’ health status. Participants were chosen because they were under mandatory isolation for COVID-19 after a positive rt-PCR result was detected during a national sampling for SARS-CoV-2. During sample collection, the most prevalent circulating variants were A23.1 and Delta. Dates of infection (Day 0) were calculated using the initial rt-PCR-confirmed COVID-19 diagnosis and, if available, the date of the first hospitalization. Participants were contacted weekly for the first month, then monthly for the next 28 months. Volunteers reflected typical hospital admissions at the time, consisting of a spectrum of mild and largely asymptomatic illnesses with a one-day median gap between PCR and admission dates (IQR, 1-3). During the follow-up, we gathered participant demographics, clinical complaints, and complete blood counts.

Negative controls included specimens collected between 2012 and 2017, prior to the outbreak. PCR-negative suspects (SUS) and non-cases (NC) recruited simultaneously at the outbreak’s onset were utilized to establish baseline cross-reactivity. The suspects were PCR-negative individuals isolated due to past close interaction with a PCR-verified COVID-19 case. Participants who had no known prior contact with a COVID case were categorized as “non-cases.” Suspects were followed on days 0, 4, 7, 14, 20, 21, and 28 between June 10, 2020, and November 5, 2020, while non-contacts were tracked for 35 days on days 0, 7, 14, 21, 28, and 35 days after the initial PCR, between June 17, 2020, and September 21, 2020. All study procedures were approved by the Research and Ethics Committee of the Uganda Virus Research Institute (GC/127/833) and the Uganda National Council for Science and Technology (HS637ES). Participants gave their written informed consent to take part in the study. Between May 11, 2020, and May 24, 2022, 320 participants aged 14–87 years (median 31, IQR [25–37]) were recruited and followed for up to 837 days (median 155, IQR [58–277]). Two individuals lacked age information: 245 were males and 75 were females. 47 were symptomatic, 192 were asymptomatic, and 81 did not have admission information. A maximum of three symptoms were recorded per individual; 15, 19, and 13 participants, respectively, exhibited one, two, and three symptoms. The most common symptoms were cough, fever, and headache, as shown in **Table 1**.

**Table 1:** Summary of recorded admission symptoms.

Symptom	n
Cough	17
Fever	16
Headache	16
Runny Nose	11
Sore Throat	8
Chest Pain	5
Shortness of Breath	3
General Weakness	2
Flu	2
Loss of Smell	2
Severe Abdominal Pain	2
Sneezing	1
Mild Headache	1
Blocked Nostrils	1
Loss of Taste	1
Chills	1
Feels hotter	1
Sharp Pain Around Arm	1
Slight Cough	1
Total	47

The table shows the frequency of symptomatic participants per registered symptom at the time of initial admission among the 47 symptomatic participants.

### Study specimens

The median duration between diagnosis and the collection of the first plasma sample from 320 patients was one day (IQR 1 to 3). A total of 2,430 plasma samples were analysed. Overall, 225/320 (70%) participants had at least four longitudinal samples, allowing studies of the development and persistence of antibody responses against SARS-CoV-2, regardless of re-infection. In addition, we assessed fifty non-contacts (NC) and fifty-four suspects (SUS).

### Conventional in-House ELISA for detection of anti-SARS-CoV-2 binding antibodies

Spike-, RBD-, and N-directed IgG, IgM, and IgA antibodies were quantified using an in-house ELISA adapted from Pickering et al. (37), optimized and validated for use in this largely asymptomatic or mildly symptomatic COVID-19infected population (38). Briefly, 96-well flat-bottomed medium-binding plates (Greiner Bio-One, #655001) coated with 50 μl of N-, RBD-, or S-Protein antigens based on the wildtype prototype strain (R&D Systems #10474-CV-01M, #10549-CV-01M) at three µg/ml (0.15µg per well) in PBS were incubated overnight at 4°C. The plates were then washed 5x with 0.01M PBS containing 0.05% Tween 20 (PBS-T) with a BioTek 405 TS microplate washer and blocked with PBS-T containing 1% BSA (Sigma, #A3803) for 1 hour at RT. Heat-inactivated (56˚C for 30 mins) plasma/serum samples diluted at 1:100 in PBS-T with 1% BSA were added in duplicate and incubated for 2 hours at RT. Following five washes with PBS-T, horseradish peroxidase-conjugated, goat anti-human IgG (γ-chain specific, Sigma, #A0170, 1:10,000 dilution), IgM (μ-chain specific, Sigma, #A6907, 1:1,000 for S and 1:5000 for N), or IgA (α-chain specific, Sigma, #A0295, 1:1,000 dilution) detection antibodies in PBS-T containing 1% BSA was added for 1 hour at room temperature (RT). Pre-determined negative and positive plasma samples, monoclonal antibodies, CR3009 (2µg/ml) for N or CR3022 (0.1µg/ml) for S and included two sets of duplicate blank wells as controls. Finally, the wells were washed and dried by tapping on absorbent paper towels. 50 μl of 3,3′,5,5′-Tetra-methyl benzidine (TMB) substrate (Sera Care, #5120-0075) was then added for 3 minutes, followed by 50 μl of 1M Hydrochloric acid (Sera Care #5150-0021) to stop the reaction. We read the plates at 450nm with a BioTek ELx808 microplate reader using the BioTek GEN5 software. Blank well OD values were subtracted from those in specimen wells to obtain the net response. Receiver operator characteristic (ROC) analysis derived cut-offs for S-, RBD-, and N-directed IgG, IgM, and IgA optical densities were 0.432, 0.356, 0.201(S protein), 0.214, 0.350, 0.303 (RBD), and 0.395, 0.229, 0.188 (N protein), respectively, as described elsewhere (38).

### Estimating binding IgG, IgM, and IgA antibody concentrations

We diluted 10 mg/ml of purified human IgG (Sigma, #12511) and 5 mg/ml of purified IgA (Sigma, #12636) commercial standards to 4.52 and 2 mg/ml, respectively, subjected them to seven 10-fold serial dilutions ranging from 1000 to 0.001 ng/ml and incubated them together with the test samples. Purified human IgM (Sigma, # 18260) was reconstituted from 10 to 1 mg/ml and subjected to seven 5-fold serial dilutions ranging from 1000 to 0.06 ng/ml. Standards were incubated in wells pre-coated with 50µl of anti-human kappa and lambda capture antibodies (Southern Biotech, #2060-01, #2070-01, 1:1 ratio, diluted 1:500). The OD450 values of the standards were used to create a non-linear, 4-parameter logistic (4-PL) modelled standard curve using the BioTek GEN5 software. The best linear range fit of the different standard curves was used to extrapolate antibody concentrations, which were then corrected for the associated dilution factor. Concentrations less than the detection limit were assigned a value of 0 ng/ml.

### Serological inference for reinfection after primary SARS-CoV-2 infection

Reinfections are typically identified through viral genomic sequencing of nasopharyngeal swab samples (39). Here, self-reports were used to detect possible reinfections, since all the cases we recruited as incident cases. Others have used varied methods to differentiate reinfection from initial infection (40, 41). One macaque study showed a 7.6-fold rise in N-IgG antibody as indicative of reinfection (21), while a human West Africa study suggested a 7-fold rise (20); similar titre rises were also observed in studies from high-income settings (42, 43). Our serological data from two SARS-CoV-2 reinfected patients with rt-PCR confirmation showed an 11-fold rise in N-IgG antibody concentration after reinfection. Then, to strongly suggest the absence of reinfection, we applied a stricter threshold of no more than a 2-fold increase in N-IgG antibody concentration. Consequently, 24 individuals were assumed to have never been infected again, and 127 individuals were presumed to have been re-infected during the follow-up period.

### Serological inference for vaccination after primary infection

Exactly one year after reporting the first COVID-19 case, Uganda launched its first COVID-19 mass vaccination program on March 10, 2021, using the AstraZeneca (AZN) vaccine and initially targeting front-line staff. Consequently, specimens taken before March 10, 2021, or around 10 months of this cohort follow-up, are deemed vaccine naïve. Immunization was subsequently validated using vaccination certificates and, if missing, serological data. Using available full-dose AZN serological data from nine participants, we calculated a fold-rise in S-IgG ranging from 3.7 to 255.8 ng/ml (median 21.1; IQR: 9.3-31.1; mean 45.3; 95% CI; 15-98) from baseline before the first vaccination to 14 days post-boost. We then used a stricter threshold of a 2-fold rise or less in S-IgG to denote the lack of vaccination uptake. Accordingly, 12 participants with less than a 2-fold rise in S-IgG across the follow-up period were classified as never having been vaccinated.

### Missing data management

Antibody concentrations over the assay’s upper detection limit were serially titrated to achieve optimum titres, and those below detection were given a value of 0 ng/ml. Samples lacking categorical variables were excluded from analyses of those categories, and “n” was stated in the corresponding figure and table.

### Statistical analysis

Concentrations, and optical densities of S-, N-, and RBD-directed IgG, IgM, and IgA antibodies were measured over time. From categorical data, proportions were derived using descriptive analyses, while summary statistics were derived from continuous variables. Using box plots and Wilcoxon rank sum tests, ODs and concentrations by gender and symptoms were compared. Using Spearman’s rank correlation test, correlations between continuous variables were estimated. Individual profile plots were calculated to visualize the progression of antibodies for each subject. Due to the imbalanced nature of the specimen time points, locally weighted scatterplot smoothing (LOWESS) analysis was used to visualize the average antibody evolutions across time. Statistical and graphical displays were generated using R Version 4.1, STATA Version 15, and GraphPad Prism Version 9.40; p-values 0.05 were deemed statistically significant.

## Results

### Baseline clinical chemistry and comorbidities

Baseline comorbidity data was captured for 62 subjects. Diabetes (1), HIV+ (1), hypertension (1), and peptic ulcer disease (1) were identified as comorbidities in four participants. There was no statistically significant difference between antibody levels in participants with comorbidities and those without. Baseline CBC parameters were assessed in 71 participants and there was no significant difference between SARS-CoV-2 PCR-positive and negative individuals. However, given the small sample size and lack of baseline data for more participants, no definitive conclusions can be drawn as to whether or not comorbidities had an effect on antibody levels in this study. This analysis lacked the power to detect any significant differences between those with and without comorbidities.

### Males and asymptomatic cases had higher early Spike-directed antibodies

Since the initial data were collected on a weekly basis, we first evaluated the antibody formation profiles during the acute period, then examined the first two months to determine the cohort peak of the primary IgG response, and then reported the overall durability. Using 434 specimens collected from 202 people with a median age of 31 between 10 June 2020 and 27 September 2021, the first month of S- and N-directed IgG, IgM, and IgA antibody profiles were established. (IQR 25–37 years). The age of one of the 202 participants with first-month specimens was unavailable; 148 (73.27%) were males, 31 (15.35%) reported minor symptoms, and 138 (68.32%) were asymptomatic. In this group, 33 individuals who lacked admission symptom reports were eliminated from the analysis by symptoms.

**Figure 1:**
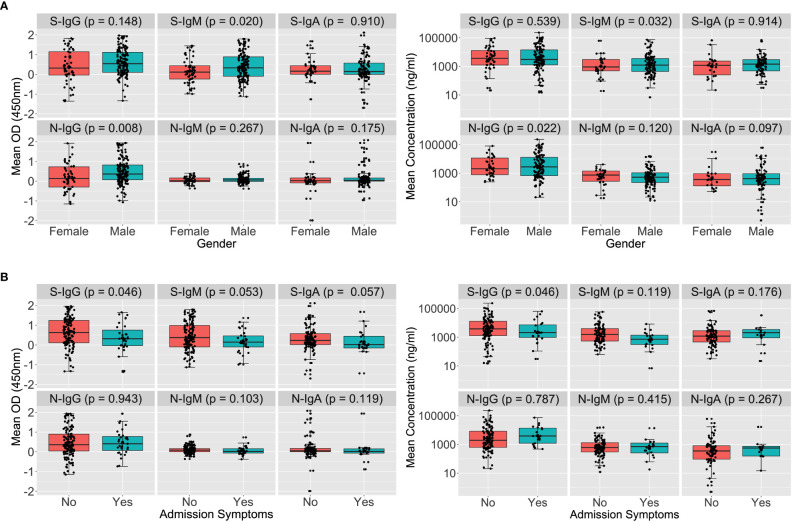
Early Spike and Nucleoprotein-directed antibodies by gender and symptoms. **Figure 1** illustrates SARS-CoV-2-specific antibody responses throughout the first month of infection using the Wilcoxon rank sum test. The optical densities and concentrations (ng/ml) of Spike- and Nucleoprotein-directed IgG, IgM, and IgA antibodies are compared, stratified by gender **(A)** and symptoms **(B)**. P-values less than or equal to 0.05 were regarded as statistically significant.

A Wilcoxon rank sum test to stratify early antibodies by gender revealed that the median S-IgM and N-IgG concentrations tended to be greater in men than in females, with p-values of 0.032 and 0.022, respectively, as seen in **Figure 1A**. Males’ concomitant high anti-Spike IgM and anti-N antibody concentrations indicate an early antigenic load-driven antibody response. S-IgG concentration was considerably greater in asymptomatic individuals than in symptomatic participants, with a p-value of 0.046 (3820; IQR 1300–12875 vs. 2120; IQR 977–7322 ng/ml [71.63; IQR 24.35-241.16 BAU/ml vs. 39.71; 18.30-137.13 BAU/ml]). Spike-directed IgM ODs, which are suggestive of the early antibody response, were shown to be greater in asymptomatic than in mildly symptomatic participants (0.373 vs. 0.146; p-value 0.053; **Figure 1B**). These findings suggest that rapid, early, and potent Spike-directed IgG, IgM, and IgA antibody responses are characteristic correlates that distinguish asymptomatic COVID-19 from COVID-19 with moderate symptoms. This emphasises the need for prompt induction of Spike-directed antibodies to control the progression of COVID-19 disease.

### Baseline Spike cross-reactivity in PCR-negative contacts and non-contacts was low

**Figure 2:**
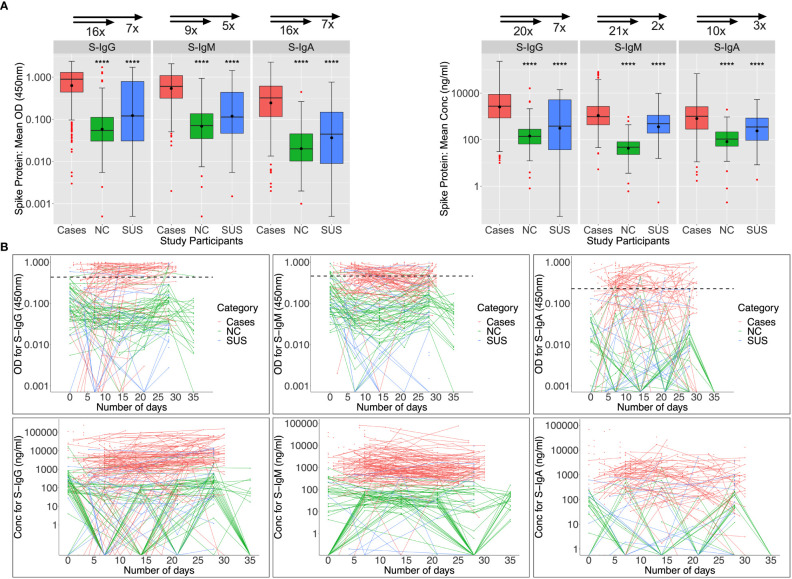
Anti-Spike antibody responses during the first month of infection. Individual subject patterns of Spike-directed IgG, IgM, and IgA antibody responses throughout the first 35 days of primary infection are shown in Figure 2. SARS-CoV-2 cases with PCR diagnosis of infection are compared with PCR-negative uninfected contacts (SUS) and non-contacts (NCs). **(A)** compares medians and interquartile ranges for antibody optical densities at 450 nm and concentrations (ng/ml) among cases, NC, and SUS individuals using box plots and the Kruskal-Wallis test. Profiles of spike-directed IgG, IgM, and IgA optical densities and concentrations are shown in **(B)** as spaghetti plots, with the graphs stratified by exposure and PCR-confirmed infection status. P-values less than or equal to 0.05 are regarded as statistically significant; ∗∗∗∗p ≤ 0.0001. Optical Density (OD) threshold values for S-IgG, S-IgM, and S-IgA were 0.432, 0.459, and 0.226, respectively.

Among 89 patients evaluated at baseline (days 0-6), Spike-directed IgG, IgM, and IgA seroconversion rates increased from 57.3%, 66.3%, and 42.7% to 88%, 66%, and 34%, by week 5 post-infection, respectively (**Figure 2A**). During the first month of the epidemic, S-IgG, -IgM, and -IgA antibody concentrations in cases were 20, 21, 10, 7, 2, and 3-fold higher than in NC and SUS, respectively. Some NC and SUS participants had detectable cross-reactive anti-Spike IgG and IgM antibodies at baseline (day 0). Three (6.38%) and seven (14.89%) of the 47 NC with baseline specimens revealed baseline cross-reactive S-IgG and -IgM antibodies. In contrast, only one (9.09%) and two (18.18%) of the eleven SUS cross-reacted, with no significant difference between NCs and SUS. There were no cross-reactive anti-Spike IgA responses at baseline, **Figure 2B**.

### Baseline N-IgM cross-reactivity in PCR-negative participants was high

**Figure 3:**
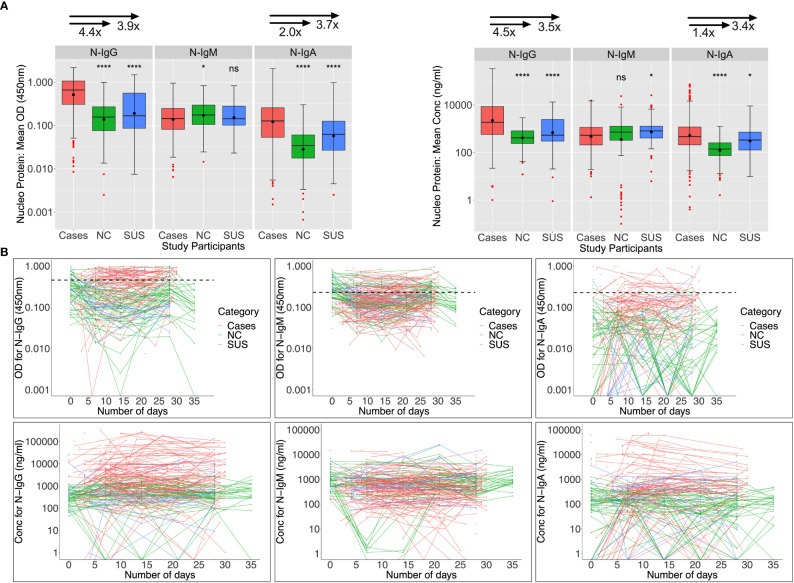
Anti-Nucleoprotein antibody responses during the first month of infection. The Nucleoprotein-directed antibodies throughout the first month of infection are summarised in Figure 3. Comparisons are made between PCR-negative, uninfected suspected contacts (SUS), uninfected non-contacts, and SARS-CoV-2 infected patients (NCs). IgG, IgM, and IgA antibody optical densities at 450 nm and concentrations (ng/ml) are compared using box plots and the Kruskal-Wallis test for cases, NC, and SUS participants **(A)**. Individual anti-spike IgG, IgM, and IgA antibody profiles are shown as spaghetti plots in **(B)**, stratified by exposure to infection and PCR-confirmed infection status from the first date of PCR or admission. Significant P-values are those that are less than or equal to 0.05; otherwise, they are not significant (ns); ns p > 0.05, ∗p ≤ 0.05, ∗ ∗ ∗ ∗ p ≤ 0.0001. The respective IgG, IgM, and IgA cut-off values for the nucleoprotein were 0.454, 0.229, and 0.225.

Nucleoprotein-directed IgG, IgM, and IgA seroconversion rates were 55.1%, 30.3%, and 25.8% among 89 infected patients assessed at baseline (days 0-6), respectively; and these rates increased to 74%, 32%, and 28% five weeks later. As anticipated, N-IgG and N-IgA antibodies at baseline were significantly higher in patients than in controls. Anti-N IgM levels were suboptimal and comparable across infected and uninfected individuals (**Figure 3A**). Among 47 NCs with day 0 specimens, cross-reactive Nucleoprotein-directed IgG (n = 13; 27.66%), IgM (30; 63.84%), and IgA (1; 2.13%) antibodies were present at baseline. Five (22.7%), thirteen (59.1%), and three (13.6%) of the eleven SUS participants had IgG, IgM, and IgA cross-reactivity at baseline, respectively. As demonstrated in **Figure 3B**, baseline -IgG and -IgM cross-reactivity with the Nucleoprotein was more prevalent among NCs than the Spike, Fisher exact test (p-values 0.006 and 0.00001, respectively). The unusual SARS-CoV-2 Nucleoprotein-specific cross-reactivity among certain SARS-CoV-2 PCR-negative individuals implies probable undiagnosed exposure at the outbreak’s onset.

### Robust anti-Spike IgG was rapidly elicited, while IgM and IgA waned early

LOWESS analysis was used to describe the chronologies of the first month of S- and N-directed IgG, IgM, and IgA antibodies in 434 samples collected from 202 individuals with a median age of 31 (IQR: 25-37 years) between June 10, 2020, and September 27, 2021. One individual had missing age information, 148 (73.27%) were males, and 54 (26.73%) were females. In terms of clinical presentation, 31 (15.35%) patients had moderate symptoms at the time of admission, 138 (68.32%) had no symptoms, and 33 (16.33%) had no symptoms reported at the time of admission. IgM and IgA were temporary and peaked early in the infection, alongside steadily rising IgG, which quickly surpassed IgM and IgA on the seventh day. Spike-directed IgM peaked at 8-12 days with a median concentration of 1229 (IQR 535-3752 ng/ml) equivalent to 45.27 (IQR; 19.82-138.44BAU/ml), while IgA peaked at 7-10 days with a median concentration of 1162 (IQR 289-2572 ng/ml) equivalent to 221.77 (IQR; 55.24-490.87 BAU/ml) and decreased at 19 days. For the Nucleoprotein, IgG surged above the cut-off after three days and subsequently increased for the duration of the month. For the duration of the first month, both IgM and IgA levels remained below the threshold (**Supplementary Figure 1**).

Considering the Spike-directed antibodies in the first two months of infection, IgM started higher than IgG, gradually dropped, and waned at 59 days. S-IgG overtook IgM by 4.5 days, reached its peak between 25 and 37 days (4612; 1569-12947 ng/ml [86.38; 29.47-242.57 BAU/ml]), then began to decline while continuing to be over the cut-off. Regarding the Nucleoprotein, IgG levels were consistent until day 36 (1801.9; 543.5-7926.9 ng/ml), after which they declined for the remainder of the research period. In contrast, N-IgM remained below the cut-off throughout the duration of the research (**Supplementary Figure 2**).

### Spike antibodies dominated and persisted longer than Nucleoprotein antibodies

The overall chronology of induced S- and N-directed antibodies across 837 days (IQR 60–287) of follow-up was then evaluated. During the period from 11 May 2020 to 20 October 2022, 2,498 specimens obtained from 320 patients were analysed. Participants’ ages ranged from 14 to 87 (median: 31; interquartile range: 25–37), with 245 men, 75 females, 47 symptomatic, 192 asymptomatic, and 81 having no symptom reports. Based on a twofold increase in N- and S-IgG to infer reinfection and vaccination, respectively, 24 people were presumptively not reinfected, and 12 participants were neither reinfected nor vaccinated over the course of this investigation. Using an 11-fold increase in N-IgG concentration, 127 participants were presumed to have been reinfected.

**Figure 4:**
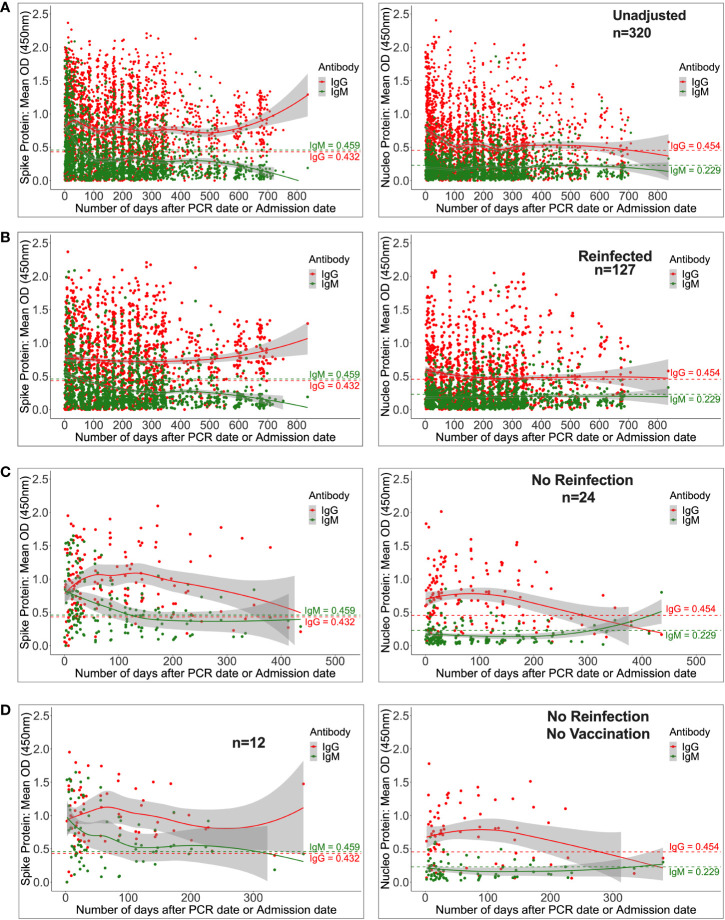
Durability of anti-Spike antibodies with or without reinfection and vaccination. **Figure 4** shows LOWESS analysis curves that summarise the longitudinal persistence of Spike-directed IgG and IgM antibodies over the duration of the study investigation. Antibody optical densities at 450 nm and concentrations (ng/ml) for 320 individuals **(A)** with moderate or asymptomatic original COVID-19 illness with **(B)** or without downstream reinfection **(C)** and vaccination **(D)** are shown. The broken horizontal lines represent seropositivity cut-offs for IgG (red) and IgM (green) antibodies. The data is more reliable where the confidence intervals are smaller.

Regardless of reinfection or immunization status, Spike-directed IgG peaked between 25 and 37 days and was greater and more durable than N-IgM. From 500 days on, there was an apparent increase in IgG (11524; 2453-32438 ng/ml [215.91; 46.032-607.61 BAU/ml]), presumably owing to downstream reinfections and vaccines, as well as the large confidence interval due to the few final data sets. Concurrently, IgM levels gradually decreased and faded after 59 days (**Figure 4A**). Initial IgG levels were stable among the 127 reinfected participants, with OD values more than the threshold. Throughout the study, IgG gradually increased at 500 days, but S-IgM decreased at 56 days (**Figure 4B**). There was a quick increase in IgG levels among the 24 people who were never reinfected; IgG levels surpassed IgM after just three days, peaked between 125 and 138 days (5236; IQR 3218-10185 ng/ml [98.15; 60.35-190.84 BAU/ml]), and subsequently steadily decreased. At 122 days, S-IgM levels declined and faded (**Figure 4C**). For the 12 individuals who were never vaccinated or reinfected, IgG exceeded IgM by five days, peaked between 64 and 69 days (4665; interquartile range [IQR]: 4665–4665 ng/ml [87.37; 87.37-87.37 BAU/ml]), and subsequently fell progressively during the remainder of the follow-up period. Contrarily, S-IgM decreased 358 days after the first infection (**Figure 4D**).

Considering the Nucleoprotein, IgG levels were initially high, then declined and finally disappeared by day 698, whereas N-IgM levels remained undetectable throughout (**Supplementary Figure 3A**). The mean N-IgG level among the 127 reinfected participants was 1012 (interquartile range [IQR] 305.5–2880 ng/ml [11.87; 3.64-33.55 BAU/ml]) after 245 days, and subsequently it remained stable at that level for the remainder of the research. For the duration of the study, N-IgM never exceeded the threshold (**Supplementary Figure 3B**). Among the 24 people who were never reinfected, N-IgG levels were highest between days 75 and 95 (5816.5; 2309.8-10046.6 ng/ml [67.84; 27.0-117.13 BAU/ml]), and then gradually declined over the next 295 days (**Supplementary Figure 3C**). N-IgG levels in 12 people who were never re-infected or vaccinated dropped after 276 days but N-IgM levels remained below the cut-off level the whole time (**Supplementary Figure 3D**).

Anti-Nucleoprotein antibodies are a primary target of current surveillance tests, and 48 (24%) of the 202 SARS-CoV-2 PCR-confirmed cases evaluated during the first month of infection lacked detectable Nucleoprotein antibodies. There was no difference in the prevalence of this finding between mild and asymptomatic cases. Nucleoprotein seropositivity rates steadily reduced after initial infection, dropping below 50% after five months and 21% after 24 months (**Supplementary Table 1**). Collectively, our results demonstrate that in individuals with a history of moderate and asymptomatic COVID-19, Spike-directed antibodies predominate and persist longer than the Nucleoprotein-directed antibodies, and this holds true regardless of subsequent reinfection and immunization. In addition, the results suggest that the use of N-IgG for monitoring of mild and asymptomatic convalescent populations may grossly underestimate the true frequencies of past exposure.

### Spike IgG antibodies were higher than and positively correlated with anti-RBD IgG

**Figure 5:**
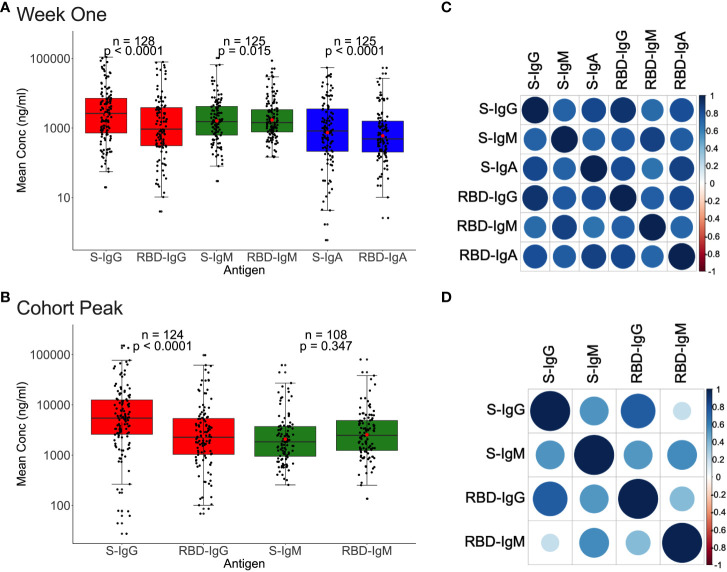
Relationships between Spike and RBD antibody concentrations. **Figure 5** demonstrates the association between whole Spike- and RBD-directed antibody concentrations during the first week **(A)** and the cohort peak **(B)** of the primary antibody response. Box plots compare Spike and RBD antibody concentration medians using a paired Wilcoxon test. Correlation plots show the pairwise Spearman’s rank correlations between entire Spike and RBD ODs during the first week of the primary antibody response **(C)** and the cohort peak **(D)**. Positive correlations are symbolized by blue, while negative correlations are indicated by red. Darker and larger circles represent stronger correlations, lighter and smaller circles represent weaker correlations, and blank squares represent insignificant correlations; p-values lee than or equal to 0.05 were deemed significant. See also **Supplementary Figure 4**.

We then analysed for correlations between Spike- and RBD-directed targeting, a known hallmark of antibody functionality (17). For the 128 first-week specimens evaluated, Spike IgG, IgM, and IgA antibody concentrations were higher (2638.7, 1532.5, and 825.5 ng/ml) than RBD concentrations (931.5, 1435.25, and 487.5 ng/ml), **Figure 5A**. Correspondingly, first-week anti-Spike OD values for IgG and IgA antibodies were also higher (0.518; IQR 0.161–0991 nm and 0.170; IQR 0.041-0.540 nm) than RDB (RBD-IgG: 0.170; IQR 0.041-0.540 nm vs. RBD-IgA: 0.084; IQR 0.033–0.234 nm), paired Wilcoxon signed-rank test, with all p-values 0.0001, **Supplementary Figure 4**). However, IgM antibody OD values were comparable across Spike and RBD (S-IgM: 0.451; IQR 0.218-0.857 nm) and 0.419; IQR 0.230-0.753 nm), respectively, and consistent with the low IgM levels observed throughout this cohort (**Supplementary Figure 4**).

A similar pattern was observed using the 124 peak specimens, with Spike IgG OD values and concentrations occurring at significantly higher levels (S-IgG: 0.672 nm and 5433.7 ng/ml) than the corresponding RBD (RBD-IgG: 0.434 nm and 2264.35 ng/ml), respectively, with p-values of 0.0001 in **Figure 5B** and **Supplementary Figure 4**. Spike and RBD antibodies positively correlated during the first week and cohort peak, Spearman’s rank correlation test, all p-values 0.0001, **Figures 5C, D**.

### Frequency and longevity of Spike IgG superseded RBD IgG levels

**Figure 6:**
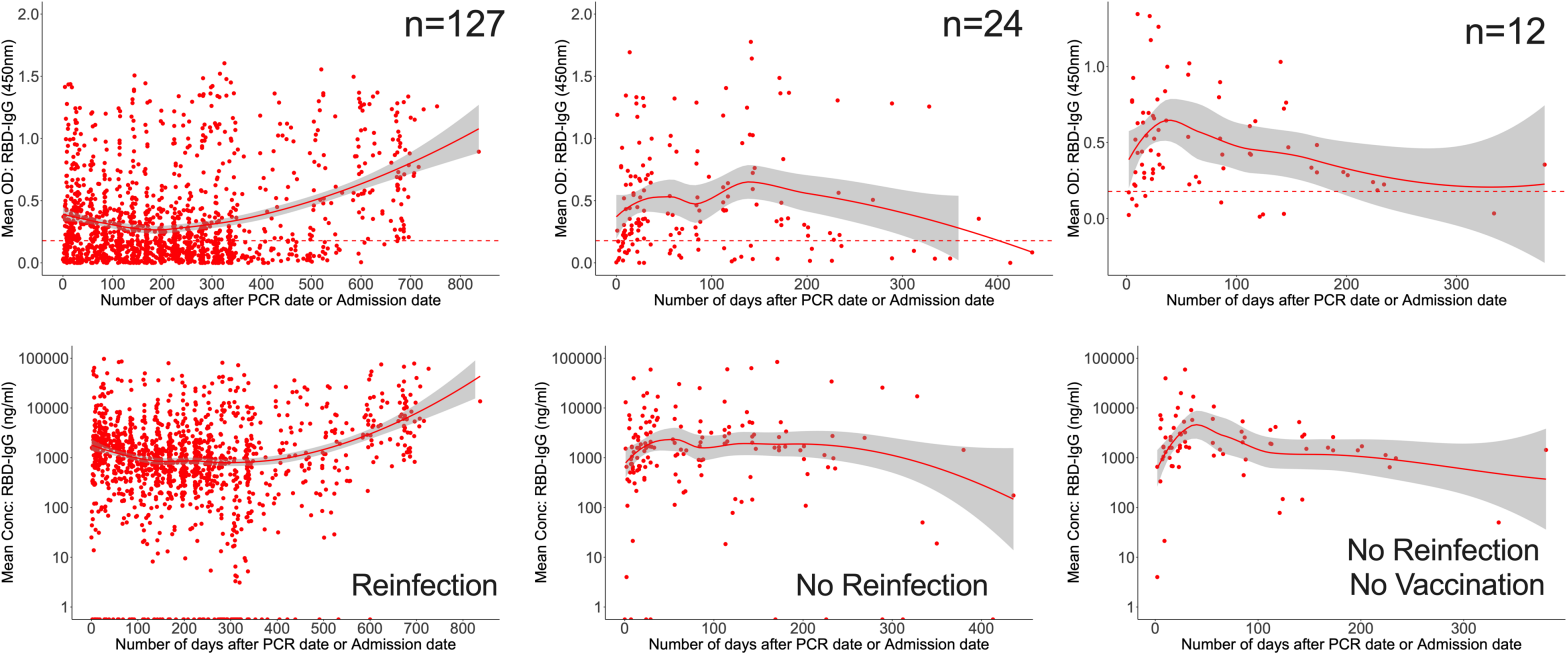
Longevity of anti-RBD antibodies. **Figure 6** illustrates LOWESS curves summarizing the dynamics of RBD-directed antibodies over time. Medians of ODs and concentrations are shown since initial infection. Dashed lines indicate cut-off points for RBD seropositivity.

Finally, we evaluated all data for the overall chronology of Spike- and RBD-directed IgG antibodies throughout the course of 28 months of cohort follow-up, irrespective of vaccination and reinfection status. The persistence of antibodies varied according to reinfection and vaccination status. For the 127 patients that were reinfected, RBD-IgG concentrations first decreased to 830.3 (355.9–1946.1 ng/ml [15.63; 6.75-36.53 BAU/ml]) at 205 days, then gradually increased. RBD-IgG peaked between 138 and 142 days (5206.2; IQR 2807.8-34145.1 ng/ml) in 24 subjects without reinfection but with some vaccinated and waned by 402 days. In 12 naturally infected unvaccinated volunteers with no reinfection, RBD-IgG peaked at 36-40 days (11317; 8535-14100 ng/ml [293.05]; 221.03-365.10 BAU/ml) then levelled out slightly above the threshold (**Figure 6**).

**Figure 7:**
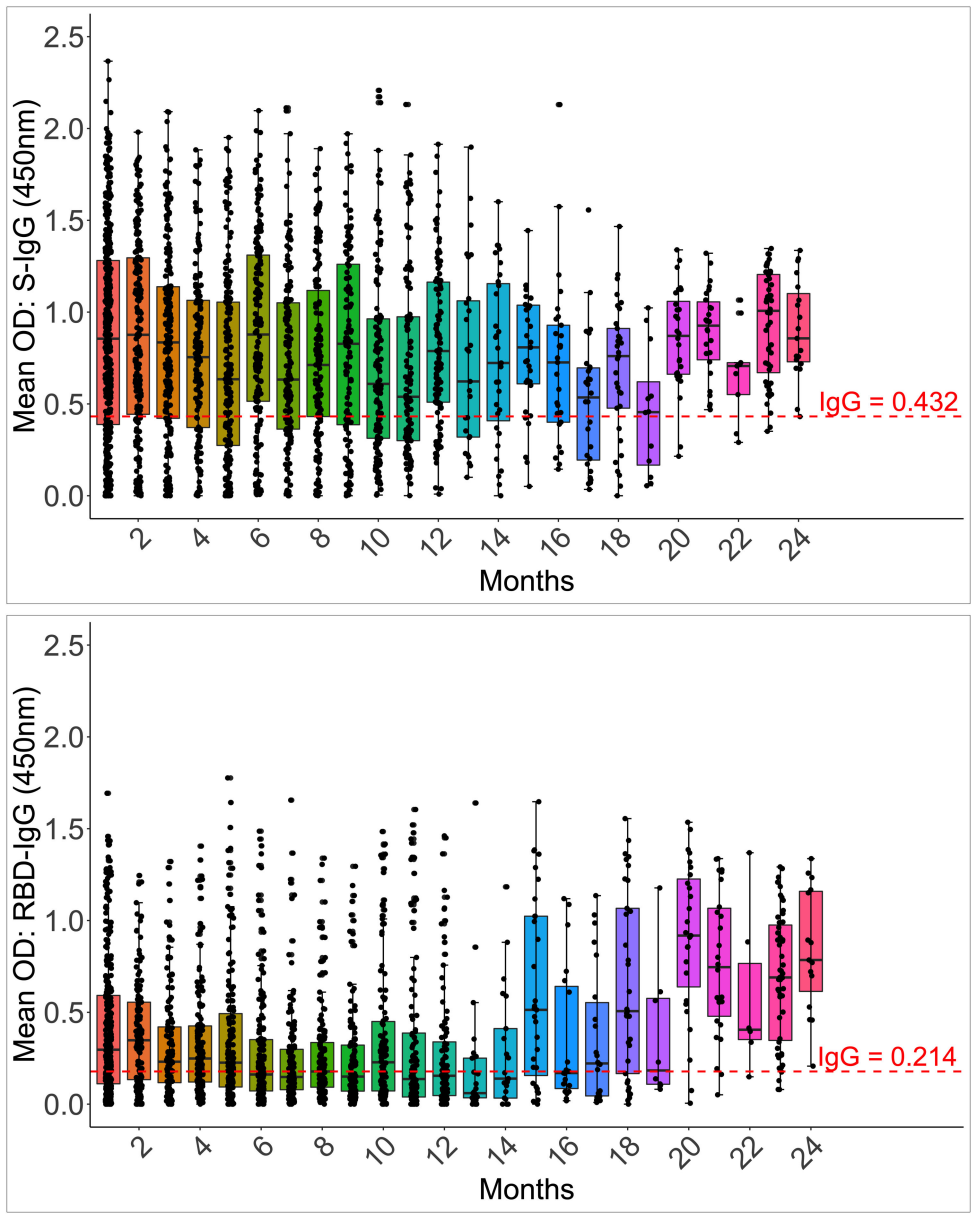
Longevity of S-IgG and RBD-IgG antibodies. **Figure 7** illustrates the frequency and longevity of S-IgG and RBD-IgG antibodies since initial infection to 24 months, post-infection. Box plots illustrate the median ODs and interquartile ranges over time. Dashed lines indicate cut-off ODs for S-IgG and RBD-IgG seropositivity.

Anti-Spike IgG antibodies were substantially greater (0.892; IQR 0.442, 1.294 450nm) than RBD IgG antibodies in the first month (0.301; 0.123, 0.611 450nm). By one month, 85.6% and 73.3% of 202 and 165 participants seroconverted to the Spike and RBD IgG, compared to 75.81% and 78.22% of 124 at the cohort peak, respectively. The median S-IgG seropositivity rates were consistently over 50% throughout. Within seven months, almost three months after the IgG primary peak (days 115–127), the percentage of seropositive individuals for RBD-IgG had declined below 50%. Spike seropositivity was greater than RBD up to 14 months; **Supplementary Table 2**. While Spike IgG OD readings stayed above the cut-off throughout, RBD OD values dropped below the cut-off after six months (**Figure 7**). With p-values of 0.05, there were greater probabilities of losing RBD than Spike IgG seropositivity for up to 16 months (**Supplementary Table 2**). The results reveal that Spike- and RBD-directed IgG antibodies can linger for up to two years following a moderate, asymptomatic case of COVID-19 illness, even if no subsequent symptomatic reinfections occur during this time. The much faster fading of RBD-directed antibodies may be indicative of diminishing functionality over time.

## Discussion

To evaluate exposure and relationships with protection, longitudinal studies of Spike- and Nucleoprotein-directed antibody levels in moderate and asymptomatic COVID-19 illness, that primarily occurred in sub-Saharan Africa, are required. This is, to our knowledge, the first and longest study to comprehensively examine the longitudinal profiles of adaptive response to mild and asymptomatic COVID-19 illness in a sub-Saharan African setting. The strength of this study lies in its early and standardised sampling of a participant group representative of the country’s initial infection with known dates of infection, which was chosen with minimal bias. This approach allowed for statistically significant comparisons and conclusions between virus-positive and virus-negative individuals. In addition, the design permitted a comprehensive assessment of the intensity and duration of immunity across waves and its decline over time. This study provides a comprehensive overview of the pandemic in a sub-Saharan African context, distinct from other geographies and for which no data exist on the evolution of immunity, yet it is required for evidence-based policy in this region. Using a validated in-house binding antibody ELISA, we describe the chronology of SARS-CoV-2 Spike-, RBD-, and Nucleoprotein-specific IgG, IgM, and IgA antibodies, including their evolution, duration, seroconversion rates, and protection associations, as the pandemic spread throughout Uganda starting in March 2020. Participants were confirmed rt-PCR SARS-CoV-2 infected Ugandans and uninfected Ugandans with extremely mild and asymptomatic COVID-19. In the first week of infection, 51 (57.3%), 59 (66.3%), and 38 (42.7%) of 89 confirmed cases seroconverted for Spike-directed IgG, IgM, and IgA seroconversion antibodies, respectively. Within a week of infection, robust Spike-directed antibodies were elicited (S-IgG: 49.50 (13.51, 134.37 BAU/ml; S-IgM: 56.61, IQR 23.20, 154.91ng/ml; and S-IgA: 157.63 (40.83, 676.58 BAU/ml), with levels peaking between 25 and 37 days (S-IgG: 4612; 1569-12947 ng/ml and S-IgM 2264.35 (1033.15, 5346.98 ng/ml). Antibodies specific to the virus were found to be much more robust in asymptomatic than in symptomatic infection, with strong Spike-directed IgG, IgM, and IgA antibodies dominating early in the course of the infection.

The clinical significance of antibody responses in COVID-19 disease is still debated. Despite effective disease control, asymptomatic patients had far less SARS-CoV-2-specific antibodies than symptomatic patients in some settings, which might be linked to the higher antigenic load in symptomatic disease (44). Mild and asymptomatic infections in China were linked to a faster decline in virus-specific antibodies (45), suggesting a less durable immunity. Antibodies were found in only a few asymptomatic participants in Europe (25). This study in a sub-Saharan African setting shows that asymptomatic infection induces faster and higher levels of anti-Spike antibodies, demonstrating the importance of early Spike targeting and reducing disease severity. Our findings are consistent with the time-dependent correlations described by others (46). We show distinctive immune protective correlates distinguishing asymptomatic from symptomatic COVID-19.

Because the dates of initial positive PCR-test testing were known, Spike and Nucleoprotein IgG, IgM, and IgA antibody trajectories could be established. The early peaking of Spike-directed IgM and IgA antibodies at 9 and 10 days and their declining at 87 and 21 days, respectively, guide the interpretation of serosurveillance findings in this setting and are consistent with previous cohorts that demonstrated the simultaneous and early appearance of both antibody subsets (45). Overlapping of S-IgM with PCR negativity suggests a reasonable proxy for virus clearance in resource-limited, largely asymptomatic settings, which starkly contrasts with symptomatic, hospitalized patients in whom IgM remained detectable long after recovery (47). The early dominance of S-IgG and rapid waning of S-IgM observed here, with S-IgG surpassing S-IgM within five days, reveal early affinity maturation and isotype class switching of IgM to IgG, a known good prognostic marker in COVID-19 (48).

Antibodies to the SARS-CoV-2 Nucleoprotein can predict past infection (49), making it a primary target in many serosurveillance studies. The loss of N-IgG seropositivity in over half of the population after seven months, regardless of vaccination or reinfection status, and the absence of measurable N-IgM levels across the board suggests that the use of these markers to predict past infection may dramatically underestimate exposure rates. Anti-Spike antibodies were shown to be higher and more persistent than anti-Nucleocapsid antibodies, just as they are in non-Sub-Saharan African settings (50, 51). Anti-Spike IgG antibodies continued to rise for the duration of the study period possibly due to vaccination and transient, clinically undetectable reinfections. S-IgG levels maintained and even rose from 234 days, but S-IgM levels stayed for just 59 days. In contrast to other geographical locations where Nucleocapsid detection rates were lower in asymptomatic patients (22, 52), we found no difference in N-directed IgG between mild and asymptomatic infection, implying that N-IgG might not be a criterion for differentiating disease severity in this SSA setting. Baseline antibody cross-reactivity was present in both SARS-CoV-2 rt-PCR-confirmed negative contacts and non-contacts at the onset of the pandemic, indicating that some degree of undetected exposure occurred in both groups. The unusually high baseline N-IgM cross-reactivity in non-cases (64%) and suspects (59%) was indicative of abortive infection, as has been suggested in other settings (53).

Antibodies that target RBDs are hallmarks of protection (54, 55). Here, in a population with spontaneous infection, reinfection, and vaccination, monthly Spike antibodies were positively correlated with the respective RBD antibodies up to 14 months, indicating that protection is likely to be durable. Under natural infection, RBD-IgG peaks around 36–40 days after the initial infection and then declines to just above the threshold at roughly 9.5 months, providing useful information for efforts to boost immunization. The higher frequency and longer duration of Spike-than-RBD-directed IgG antibodies suggests that binding to other areas beyond the RBD contributes considerably to the maintenance of Spike-directed immunity in this population. While severe illness cohorts correlated antibody persistence with older age, male gender, and severe disease (23), we found no age relationships, which could be attributed to the younger cohort (31; IQR 25-37 years) studied here. We found significantly higher S-IgG levels in men early in the infection, but this coincided with high anti-N antibodies, indicating an antigenic load-driven early response. Beyond the initial response, no gender differences were discernible. We showed that even when the disease is mild and symptomatic, humoral responses to SARS-CoV-2 can persist for a very long time.

Our study has some limitations. To begin with, the impact of age, gender, disease severity, and underling conditions conditions such as HIV, hypertension, and diabetes on the evolution of SARS-CoV-2 antibody responses has been reported before (56–58). In this study, the small sample size and the lack of baseline clinical data for most participants meant that no definitive conclusions could be drawn about whether or not comorbidities affected antibody levels. More studies are needed with larger sample sizes and more detailed baseline clinical information to draw more meaningful conclusions about comorbidity effects on antibody levels. Second, these data were collected during the initial outbreak, with follow-ups during subsequent waves. The virus has evolved, and new variants have emerged, with Omicron currently dominating (59–62); this threatens the effectiveness and recognition of previous antibodies (63–65). Efforts are underway to evaluate the antibody recognition of circulating strains and the effectiveness of prior vaccination or natural infection antibodies in neutralising circulating viruses. Third, the known correlation between RBD antibodies and viral neutralisation established elsewhere (66) suggests that these antibodies provide early and long-term immune protection. To confirm their functionality, it is necessary to evaluate their ability to neutralise circulating virus strains. Additional studies are planned to establish the precise nature of the immunological memory associated with the SARS-CoV-2 spike protein, including its functionality and durability over time and across virus variants. Another limitation is that exposure to viral antigens, whether through natural infection, vaccination, or both, is an important driver of antibody persistence. We cannot rule out the possibility of undetected re-infections occurring during the follow-up period. Using estimates of nucleoprotein antibody concentrations from other contexts (42, 67) we observed substantial increases in N-IgG antibody concentrations, suggesting the possibility of reinfection.

Additional factors, such as the introduction of vaccines and boosters, nosocomial exposures, which are important in maintaining antibody levels (68, 69), may have also contributed to the long antibody persistence. There is a need for additional research into the re-infection rate and the effect of vaccines and boosters on antibody levels. The study was also limited by the uneven gender distribution due to the predominance of males in the commercial truck driver profession. More research will be required to adequately assess gender-related determinants in this setting. Finally, while the median age of these individuals is 31 years, antibody evolution may occur at a different rate in older populations. Overall, the data is representative of Sub-Saharan Africa’s broader demography, whose median age is 19.7 years, but it highlights the need for additional research into the immune response among older populations, as this is an area of particular concern for public health due to their higher risk of severe COVID-19 outcomes.

In conclusion, we have shown that a Spike-directed humoral response to SARS-CoV-2 was formed and maintained in recovered mild and asymptomatic Ugandans, strongly suggesting the formation of lasting immunological memory that may contribute to herd immunity. A faster and more robust antibody response in asymptomatic infection suggests that those who do not experience symptoms may still have strong immunity, protecting them from progression to severe disease. Vaccines could be developed to take advantage of the strong immunological response in asymptomatic infection, reducing disease severity. Anti-SARS-CoV-2 antibody analysis can be complicated by vaccine uptake, reinfection, and diminishing antibody levels over time. Defining IgG, IgM, and IgA antibody longevity against Spike and Nucleoprotein antigens in this context with or without reinfection and vaccination provides data to guide vaccination, boosting strategies, and interpretation of serosurveillance in this and other comparable settings.

## The COVID-19 Immunoprofiling Team

Patricia Namubiru, Hermilia Christine Akoli, Susan Mugaba, Amina Nalumansi, Geoffrey Odoch, Kibengo Freddie, Deus Mwesigwa, Joseph Ssebwana Katende.

## Data availability statement

The raw data supporting the conclusions of this article will be made available by the authors, without undue reservation.

## Ethics statement

All study procedures were approved by the Research and Ethics Committee of the Uganda Virus Research Institute (GC/127/833) and the Uganda National Council for Science and Technology (HS637ES). The patients/participants provided their written informed consent to participate in this study.

## Author contributions

Conceptualization and methodology: JSer. Laboratory investigation: JSem, LK, GKO, CB, GO, JK and The COVID-19 Immunoprofiling team. Data curation, software, and formal statistical analysis: VA, TL and JSer. Resources: CN, MJ, NO and MMuw. Clinical Management of study cohort: CN, MJ, NO and MMuw. Writing- original draft; JSer, GKO and VA. Writing- reviewing and editing; PK, JF, and MC. Funding acquisition; MMus, JSer, JF, MC. Project administration: JSer and BA. Supervision; PK. All authors contributed to the article and approved the submitted version.

## Supplementary material

Supplementary Figure 1 Chronology of IgG, IgM, and IgA antibody trends during the first month of infection. **Supplementary Figure 1** shows Locally Weighted Scatter Plot Smoothing (LOWESS) curves summarising the profiles of the development of Spike-directed IgG and IgM antibodies. IgG, IgM, and IgA antibody optical densities **(A)** and concentrations **(B)** are indicated for 202 cases during their first 30 days of infection. Broken horizontal lines indicate IgG (red) and IgM (green) seropositivity cut-offs.

Supplementary Figure 2 Chronology of IgG, IgM, and IgA antibody trends during the first two months of infection. **Supplementary Figure 2** shows Locally Weighted Scatter Plot Smoothing (LOWESS) curves summarising the profiles of the development of Nucleoprotein-directed IgG and IgM antibodies. IgG, IgM, and IgA antibody optical densities **(A)** and concentrations **(B)** are indicated for 269 cases during their first two months of infection. Broken horizontal lines indicate IgG (red) and IgM (green) seropositivity cut-offs.

Supplementary Figure 3 Durability of Nucleoprotein IgG and IgM antibodies over time. **Supplementary Figure 3** shows LOWESS analysis curves that summarise the longitudinal persistence of Nucleoprotein-directed IgG and IgM antibodies over the duration of the study investigation. Antibody optical densities at 450 nm and concentrations (ng/ml) for 320 individuals **(A)** with moderate or asymptomatic primary COVID-19 illness with **(B)** or without downstream reinfection **(C)** and vaccination **(D)** are shown. The broken horizontal lines represent seropositivity cut-offs for IgG (red) and IgM (green) antibodies.

Supplementary Figure 4 Spike and RBD ODs during the first week and cohort peak time-points. **Supplementary Figure 4** demonstrates the association between whole spike- and RBD-directed antibody optical densities (nm) during the first week (**Figure 4A**) and the cohort peak (**Figure 4B**) of the primary antibody response. Box plots compare spike and RBD antibody concentration medians using a paired Wilcoxon test. Correlation plots show the pairwise Spearman’s rank correlations between entire spikes and RBD ODs during the first week of the primary antibody response (**Figure 4C**) and the cohort peak (**Figure 4D**). Positive correlations are symbolized by blue, while negative correlations are indicated by red. Darker and larger circles represent stronger correlations, lighter and smaller circles represent weaker correlations, and blank squares represent insignificant correlations; p-values of 0.05 were deemed significant.

Supplementary Table 1 Proportions of nucleoprotein IgG seropositivity over time.

Supplementary Table 2 Proportions of Spike and RBD seropositive individuals. OR (Odds ratio), IQR (Interquartile range).
