# Supplementary material for: Rapid, early, and potent Spike-directed IgG, IgM, and IgA distinguish asymptomatic from mildly symptomatic COVID-19 in Uganda, with IgG persisting for 28 months
Source: Front Immunol. 2023 Mar 16;14:1152522. doi: 10.3389/fimmu.2023.1152522 (PMC10060567; doi:10.3389/fimmu.2023.1152522)
Supplement: Supplementary Figure 1 — Chronology of IgG, IgM, and IgA antibody trends during the first month of infection. Supplementary Figure 1 shows Locally Weighted Scatter Plot Smoothing (LOWESS) curves summarising the profiles of the development of Spike-directed IgG and IgM antibodies. IgG, IgM, and IgA antibody optical densities (A) and concentrations (B) are indicated for 202 cases during their first 30 days of infection. Broken horizontal lines indicate IgG (red) and IgM (green) seropositivity cut-offs. [file Presentation_1.pdf]

Supplementary Material

Supplementary Figures

***Supplementary Figure 1**: Chronology of IgG, IgM, and IgA antibody trends during the first month of infection*

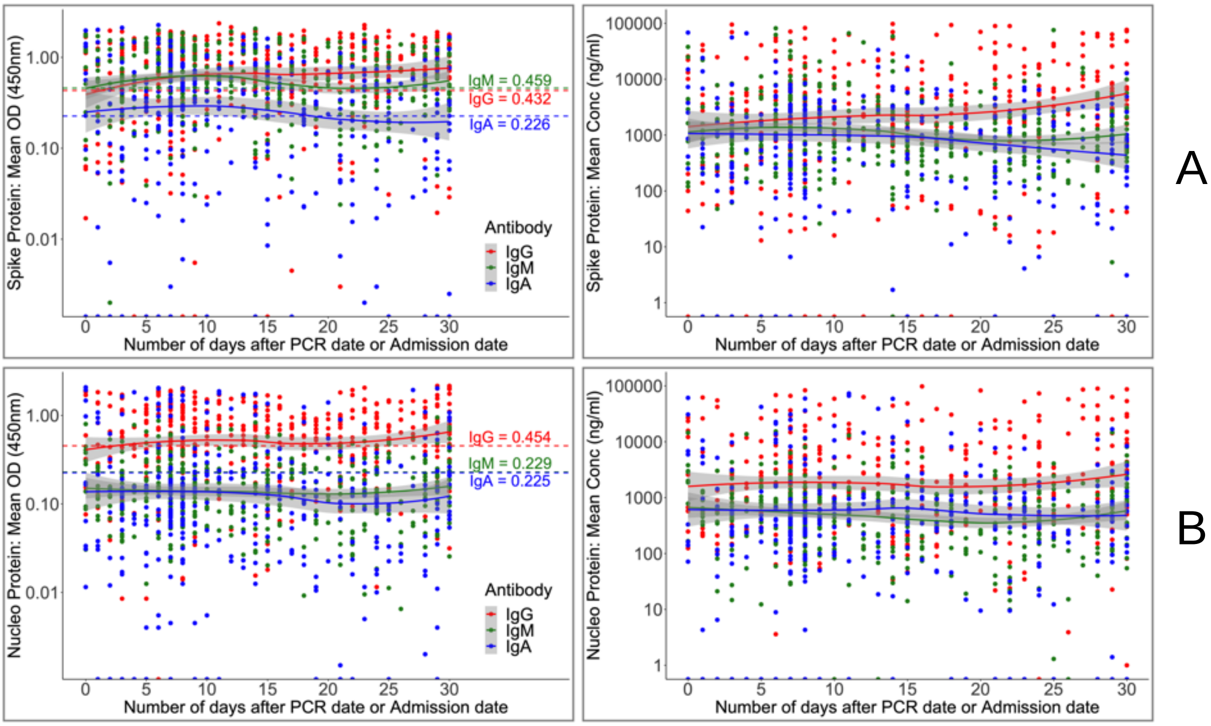

***Supplementary Figure 1** shows Locally Weighted Scatter Plot Smoothing (LOWESS) curves summarising the profiles of the development of Spike-directed IgG and IgM antibodies. IgG, IgM, and IgA antibody optical densities (**Supplementary Figure 1A**) and concentrations (**Supplementary Figure 1B**) are indicated for 202 cases during their first 30 days of infection. Broken horizontal lines indicate IgG (red) and IgM (green) seropositivity cut-offs.*

Supplementary Figure 2*: Chronology of IgG, IgM, and IgA antibody trends during the first two months of infection*

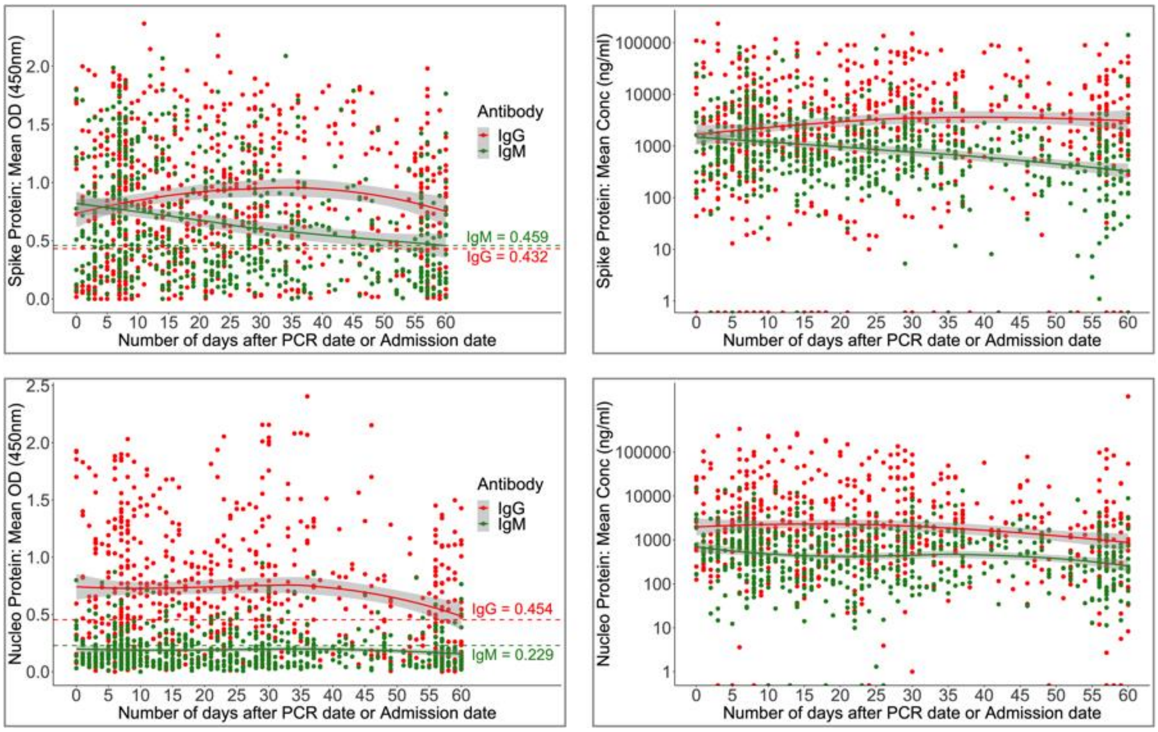

A

B

Supplementary Figure 2 *shows Locally Weighted Scatter Plot Smoothing (LOWESS) curves summarising the profiles of the development of Nucleoprotein-directed IgG and IgM antibodies. IgG, IgM, and IgA antibody optical densities (****Supplementary Figure 1A****) and concentrations (****Supplementary Figure 1B****) are indicated for 269 cases during their first two months of infection. Broken horizontal lines indicate IgG (red) and IgM (green) seropositivity cut-offs.*

***Supplementary Figure 3**: Durability of Nucleoprotein IgG and IgM antibodies over time*

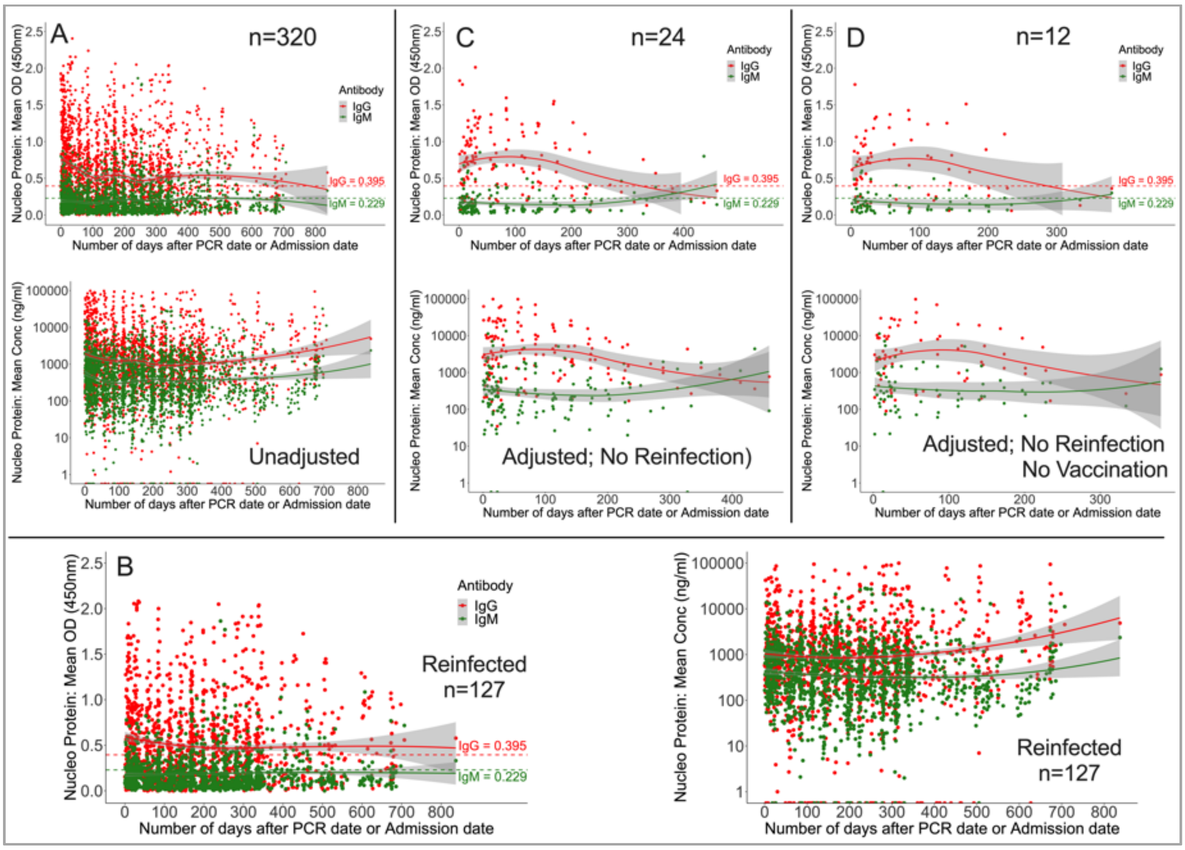

***Supplementary Figure 3** shows LOWESS analysis curves that summarise the longitudinal persistence of Nucleoprotein-directed IgG and IgM antibodies over the duration of the study investigation. Antibody optical densities at 450 nm and concentrations (ng/ml) for 320 individuals (**Supplementary Figure 3A**) with moderate or asymptomatic primary COVID-19 illness with (**Supplementary Figure 3B**) or without downstream reinfection (**Supplementary Figure 3C**) and vaccination (**Supplementary Figure 3D**) are shown. The broken horizontal lines represent seropositivity cut-offs for IgG (red) and IgM (green) antibodies.*

Supplementary Figure 4: *Spike and RBD ODs during the first week and cohort peak time-points*

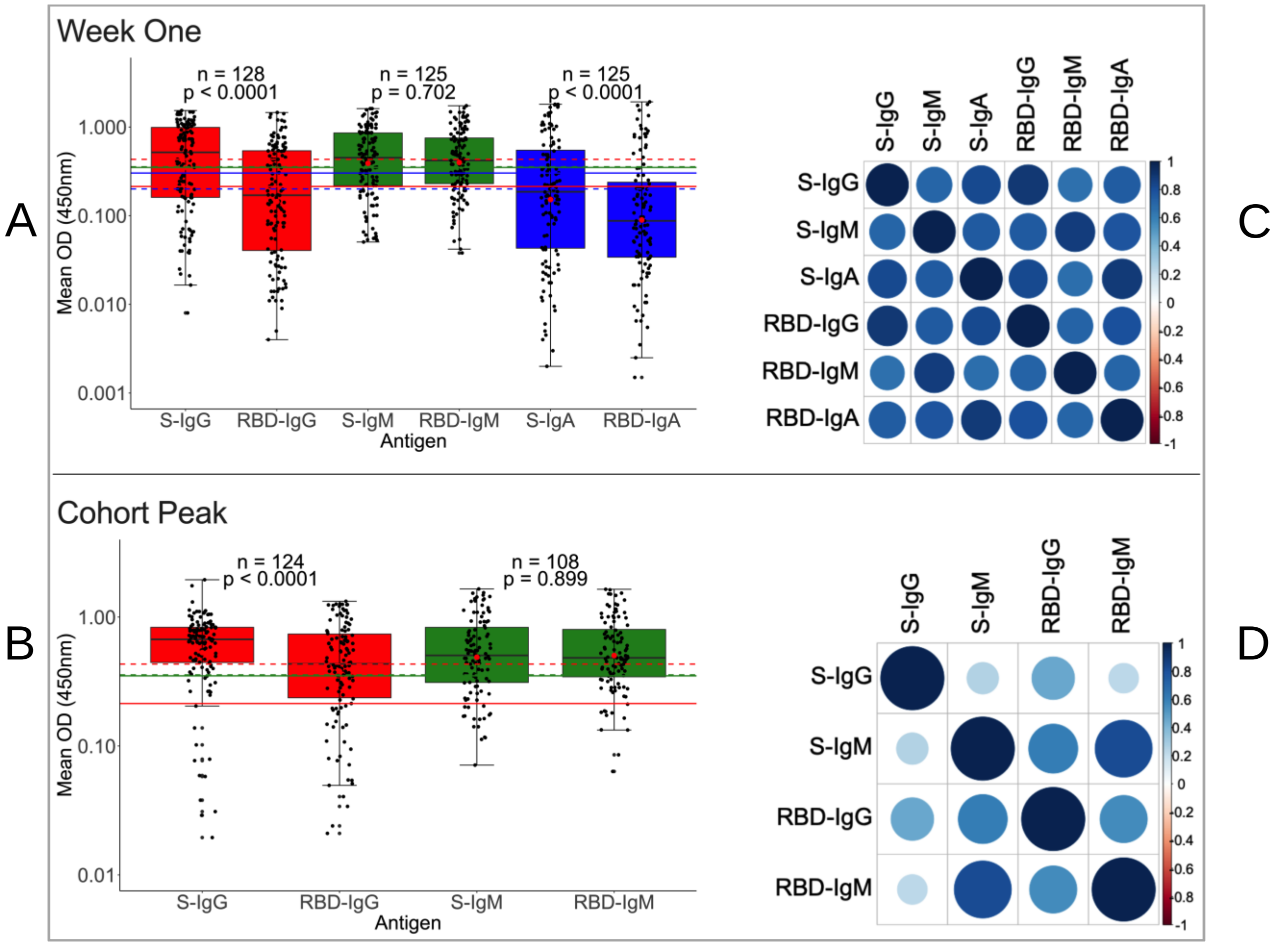

Supplementary Figure 4 *demonstrates the association between whole spike- and RBD-directed antibody optical densities (nm) during the first week (**Figure 4A**) and the cohort peak (**Figure 4B**) of the primary antibody response. Box plots compare spike and RBD antibody concentration medians using a paired Wilcoxon test. Correlation plots show the pairwise Spearman's rank correlations between entire spikes and RBD ODs during the first week of the primary antibody response (**Figure 4C**) and the cohort peak (**Figure 4D**). Positive correlations are symbolized by blue, while negative correlations are indicated by red. Darker and larger circles represent stronger correlations, lighter and smaller circles represent weaker correlations, and blank squares represent insignificant correlations; p-values of 0.05 were deemed significant.*

Supplementary Tables

***Supplementary Table 1**: Proportions of nucleoprotein IgG seropositivity over time*

Month	#N-IgG+/n (%)
Month 1	154/202 (76.24%)
Month 2	103/160 (64.38%)
Month 3	103/179 (57.54%)
Month 4	74/137 (54.01%)
Month 5	67/155 (43.23%)
Month 6	102/160 (63.75%)
Month 7	54/139 (38.85%)
Month 8	75/149 (50.34%)
Month 9	50/122 (40.98%)
Month 10	50/118 (42.37%)
Month 11	49/107 (45.79%)
Month 12	58/99 (58.59%)
Month 13	9/24 (37.50%)
Month 14	16/29 (55.17%)
Month 15	11/30 (36.67%)
Month 16	15/28 (53.57%)
Month 17	13/28 (46.43%0
Month 18	14/32 (43.75%)
Month 19	3/12 (25.00%)
Month 20	1/26 (3.85%)
Month 21	15/26 (57.69%)
Month 22	1/9 (11.11%)
Month 23	10/56 (17.86%)
Month 24	4/19 (21.05%)

***Supplementary Table 2**: Proportions of Spike and RBD seropositive individuals*

Month	Variable	Median Conc in ng/ml (IQR)	Median Conc in BAU/ml (IQR)	#+/n (%)	OR	p-values
Month 1	S-IgG	2758.7 (861.54, 8830.43)	51.748 (16.216, 165.466)	173/202	2.16	0.0038
	RBD-IgG	1621.4 (654.35, 3677.50)	42.810 (17.775, 96.038)	121/165		
Month 2	S-IgG	3153 (1219.0, 9396.1)	59.133 (22.911, 176.06)	128/160	1.88	0.0379
	RBD-IgG	1786.9 (791.8, 3393.6)	47.094 (21.333, 88.688)	71/100		
Month 3	S-IgG	3145.65(1220.08, 5903.55)	58.995 (22.931, 110.648)	138/179	1.82	0.0317
	RBD-IgG	1189.6 (535.8, 2416.3)	31.631 (14.706, 63.388)	74/114		
Month 4	S-IgG	2576.75 (898.53, 6187.08)	48.340 (16.909, 115.958)	102/137	1.57	0.1123
	RBD-IgG	958.45 (373.78, 1996.20)	25.647 (10.511, 52.512)	87/134		
Month 5	S-IgG	2029.05 (926.63, 4927.3)	38.082 (17.435, 92.364)	104/155	1.24	0.4045
	RBD-IgG	772.2 (344.75, 1733.35)	20.826 (9.760, 45.708)	95/153		
Month 6	S-IgG	2829 (1470.18, 6572.75)	53.065 (27.615, 123.182)	132/160	4.69	< 0.0001
	RBD-IgG	803.6 (347.6, 1688.9)	21.638 (9.833, 44.557)	79/158		
Month 7	S-IgG	2387.55 (1368.6, 4861.4)	44.797 (25.713, 91.130)	98/139	2.83	< 0.0001
	RBD-IgG	1014.7 (476.43, 2949.40)	27.103 (13.169, 77.189)	63/138		
Month 8	S-IgG	2230.9(1277.33, 4696.98)	41.863 (24.003, 88.050)	111/149	2.99	< 0.0001
	RBD-IgG	825.8 (413.8, 1622.0)	22.213 (11.547, 42.825)	72/146		
Month 9	S-IgG	2407.7 (845.75, 5300.65)	45.174 (15.920, 99.356)	84/122	2.57	0.0004
	RBD-IgG	745.7 (389.25, 1635.55)	20.140 (10.912, 43.176)	54/117		
Month 10	S-IgG	1922.6 (755.90, 4970.95)	36.089 (14.238, 93.181)	79/118	1.52	0.1363
	RBD-IgG	959.2 (285.0, 3008.8)	25.667 (8.213, 78.727)	64/112		
Month 11	S-IgG	1583.1 (788.1, 4502.4)	29.730 (14.841, 84.406)	66/107	1.90	0.0273
	RBD-IgG	239.2 (39.30, 2334.55)	7.027 (1.852, 61.272)	48/105		
Month 12	S-IgG	2740.1(1409.75, 5942.00)	51.400 (26.484, 111.368)	79/99	4.00	< 0.0001
	RBD-IgG	757.4 (227.0, 1680.3)	20.442 (6.711, 44.334)	46/93		
Month 13	S-IgG	1603.4 (847.25, 3680.90)	30.111 (15.949, 69.020)	14/24	2.93	0.0852
	RBD-IgG	282.5 (118.1, 1017.9)	8.148 (3.892, 27.186)	7/22		
Month 14	S-IgG	2705.5 (921.05, 6359.95)	50.752 (17.331, 119.196)	23/29	4.12	0.0306
	RBD-IgG	1260 (458.05, 2761.25)	33.454 (12.693, 72.318)	9/19		
Month 15	S-IgG	3253.65(655.18, 13447.63)	61.018 (12.351, 251.941)	25/30	1.80	0.5321
	RBD-IgG	3375 (1077.23, 6560.20)	88.207 (28.722, 170.665)	22/30		
Month 16	S-IgG	2164.1 (918.2, 3706.0)	40.612 (17.277, 69.490)	21/28	3.24	0.0691
	RBD-IgG	754.75 (565.35, 3942.50)	20.374 (15.471, 102.898)	9/19		
Month 17	S-IgG	1357.4 (384.65, 4166.80)	25.503 (7.285, 78.120)	16/28	0.92	1.000
	RBD-IgG	1066.8 (220.23, 2829.03)	28.452 (6.536, 74.073)	13/22		
Month 18	S-IgG	1412.55(100.36, 14403.15)	26.536 (1.960, 269.837)	25/32	1.19	1.000
	RBD-IgG	3156.45 (810.35, 5703.38)	82.549 (21.813, 148.484)	24/32		
Month 19	S-IgG	821.7 (152.2, 1594.34)	15.470 (2.91, 29.941)	7/12	1.38	1.000
	RBD-IgG	871.65 (343.85, 3999.88)	23.400 (9.736, 104.384)	4/8		
Month 20	S-IgG	21430.8 (5552.70, 40887.35)	401.458 (104.077, 765.860)	24/26	1.55	1.000
	RBD-IgG	6861.65(2898.88, 12655.0)	178.469 (75.881, 328.448)	23/26		
Month 21	S-IgG	13789.3 (8370.13, 29820.98)	258.340 (156.845, 558.598)	26/26	3.18	0.6110
	RBD-IgG	4533.7 (2531.65, 12474.75)	118.203 (66.374, 323.781)	24/26		
Month 22	S-IgG	5023.2 (1900.0, 12496.1)	94.160 (35.666, 234.120)	7/9	0.72	1.000
	RBD-IgG	2323.9 (1845.1, 2371.4)	60.996 (48.601, 62.226)	5/6		
Month 23	S-IgG	20438.3 (5876.0, 41535.1)	382.869 (110.132, 777.991)	54/56	2.28	0.4215
	RBD-IgG	5956.8 (1955.28, 11504.8)	155.045 (51.453, 298.671)	47/51		
Month 24	S-IgG	21072.35 (10670.27,	394.745 (199.924, 1012.864)	18/19	0.60	1.000
	RBD-IgG	5799.8 (3108.1, 23701.58)	150.98 (81.297, 614.421)	15/15		

OR (Odds ratio), IQR (Interquartile range)
